# Complete Genome Sequences of Two Mutacin-Producing Streptococcus mutans Strains, T8 and UA140

**DOI:** 10.1128/MRA.00469-20

**Published:** 2020-06-11

**Authors:** Indranil Biswas

**Affiliations:** a Department of Microbiology, Molecular Genetics, and Immunology, University of Kansas Medical Center, Kansas City, Kansas, USA; University of Maryland School of Medicine

## Abstract

Streptococcus mutans is known to produce various antimicrobial peptides called mutacins. Two clinical isolates, T8 and UA140, are well characterized regarding their mutacin production, but genome sequence information was previously unavailable. Complete genome sequences of these two mutacin-producing strains are reported here.

## ANNOUNCEMENT

Streptococcus mutans is an oral pathogen associated with human dental caries formation (1). The oral cavity harbors a complex microflora that contains ∼700 different bacterial species; nearly 30% of the microflora belongs to the genus *Streptococcus* (2, 3). To maintain its dominant presence in the oral cavity, S. mutans secretes small antibacterial peptides called mutacins to inhibit the growth of other streptococci and competing bacteria. Mutacins are classified into two categories, namely, lantibiotics, which are peptides containing the unusual amino acids lanthionine and dehydrated amino acids, and nonlantibiotics, which are unmodified linear peptides. Production of mutacin-like peptides is highly variable among S. mutans strains (4–6).

Among the few S. mutans strains from which mutacins were first purified and characterized are strains T8 and UA140 (7–9). While T8 predominantly produces lantibiotics (mutacin II), UA140 produces both lantibiotics (mutacin I) and nonlantibiotics (mutacin IV) (7–9). UA140 is a well-characterized strain that is widely used for physiological study, but its genome sequence had not been determined previously (10–13). The T8 strain is primarily used for production and characterization of mutacin II (7, 14–18), and its genome sequence had not been determined previously. The genome sequences of these two strains were determined.

The strains, which were collected long ago, were grown overnight at 37°C in Todd-Hewitt medium (BBL) supplemented with 0.2% yeast extract, under microaerophilic conditions. Genomic DNA was isolated using a MasterPure DNA purification kit (Lucigen) as described previously (19–21). DNA quantity and quality were checked with a NanoDrop spectrophotometer (Thermo Fisher Scientific) and gel electrophoresis, respectively. SMRTbell DNA libraries were prepared using the Express template preparation kit v2.0 (Pacific Biosciences) according to the manufacturer’s protocol. Samples were pooled into a single multiplexed library and size selected using BluePippin (Sage Sciences) according to the manufacturer’s recommendations. The size-selected SMRTbell libraries were annealed, bound, and sequenced on a Sequel II system with Sequel II chemistry v1.0 at SNPsaurus. Raw reads were converted to the fasta format with SAMtools (22). Flye v2.4.1 (23) with default parameters was used to assemble the T8 and UA140 genomes from 640,000,860 bases (*N*_50_, 12,098 bp) and 383,461,336 bases (*N*_50_, 13,094 bp), respectively. The final genome coverages for T8 and UA140 were 326-fold and 195-fold, respectively. The genome annotation was carried out using the IGS Prokaryotic Annotation Pipeline at the Institute of Genome Sciences at the University of Maryland (24). The T8 and UA140 strains harbor a single chromosome of 1,976,303 bp (GC content, 37.04%) and 2,005,049 bp (GC content, 37.04%), respectively.

The genome sequences were analyzed to predict putative biosynthetic gene clusters (BGCs), including mutacins, using the antiSMASH (25) and BAGEL4 (26) Web servers with default parameters. As expected, the T8 and UA140 genomes encoded mutacin II and mutacin I, respectively; both genomes also encoded nonlantibiotics and contained other BGC loci. The genome sequencing identified several new methylation motifs in S. mutans, in addition to the common GATC motif. These new methylation motifs are CGRAC and GGTGNGAGCG for T8 and CGCGA and TACNNNGTA for UA140. I think this study will provide useful information for comparative genomic analysis and understanding of the lantibiotic and nonlantibiotic repertoires in S. mutans.

### Data availability.

The complete genome sequences of these S. mutans strains have been deposited in GenBank under the accession numbers CP044492 (T8) and CP044495 (UA140). The GenBank assembly numbers for the genomes are GCF_008831345.1 (T8) and GCF_008831365.1 (UA140). The BioProject accession number for the genomes is PRJNA525085. The raw files were deposited in the SRA database under accession numbers SRR11812840 (T8) and SRR11812841 (UA140).
